# Impact of MWCNT Aspect Ratio on the Processing and Functional Properties of Buckypaper for EMI Shielding Applications

**DOI:** 10.1021/acsomega.5c10056

**Published:** 2026-02-12

**Authors:** Thais Ferreira da Silva, Erick Gabriel Ribeiro dos Anjos, Thiély Ferreira da Silva, Rieyssa Maria de Almeida Corrêa, Carlos Eduardo Moraes, Rui Alexandre Araújo Ribeiro, Braian Esneider Buitrago Uribe, Bruno Ribeiro, Michelle Leali Costa, Fabio Roberto Passador, Maria Conceição Jesus Rego Paiva, Edson Cocchieri Botelho

**Affiliations:** † School of Engineering, São Paulo State University (UNESP), Av. Dr. Ariberto Pereira da Cunha, 333 - Pedregulho, Guaratinguetá SP 12516-410, Brazil; ‡ Federal University of São Paulo (UNIFESP), Department of Science and Technology, Polymer and Biopolymer Technology Laboratory (TecPBio), 330 Talim St., São José dos Campos,12231-280 SP, Brazil; § School of Engineering, University of Minho, Azurem Campus, 4804−533 Guimarães, Portugal

## Abstract

Buckypaper (BP), a free-standing porous film composed of entangled carbon nanotube networks, is a promising material for lightweight and multifunctional electromagnetic interference (EMI) shielding. In this study, the effect of multiwalled carbon nanotube (MWCNT) aspect ratio on the processing, microstructure, electrical properties, and EMI shielding performance of buckypapers was systematically investigated. Two commercial MWCNTs with distinct geometries were used: short MWCNTs (S-MWCNT, aspect ratio ≈ 158) and long MWCNTs (L-MWCNT, aspect ratio ≈ 600). Buckypapers were fabricated by vacuum-assisted filtration with and without electrospun polyacrylonitrile (PAN) sacrificial mats. S-MWCNTs readily formed uniform, flexible, and self-supporting buckypapers without processing aids, whereas L-MWCNTs required sacrificial mats to enable film formation. Morphological and structural analyses (FEG-SEM, XRD, Raman spectroscopy, and N_2_ adsorption) showed that higher-aspect-ratio MWCNTs promote agglomeration and denser networks, while S-MWCNT buckypapers exhibited higher porosity and surface area (up to 205 m^2^.g^–1^). Impedance spectroscopy revealed higher electrical conductivity for S-MWCNT buckypapers prepared without sacrificial mats (≈10^–1^S.cm^–1^), whereas residual PAN significantly reduced the conductivity. EMI shielding measurements in the X-band (8.2–12.4 GHz) demonstrated excellent shielding effectiveness for S-MWCNT buckypapers, reaching values up to 36 dB at submillimeter thickness, with reflection as the dominant attenuation mechanism. These results demonstrate that MWCNT aspect ratio is a key parameter governing buckypaper processability and functional performance, offering valuable guidelines for the design of lightweight EMI shielding materials.

## Graphical abstract

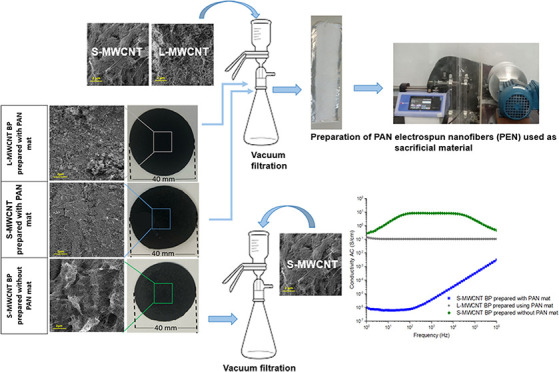

## 1 Introduction

Over the past few decades, the development of advanced materials has significantly impacted the field of structural engineering.^1–4^ Among them, nanostructured materials—particularly carbon nanotubes (CNTs) (CNTs)—have stood out due to their exceptional mechanical, thermal, and electrical properties. Since their discovery in the 1990s, CNTs have garnered increasing interest in both academia and industry.^5–9^ This attention stems from their high thermal conductivity (3000–6000 W/m·K),^10,11^ excellent electrical conductivity (10^6^–10^7^ S/m),^12,13^ and outstanding mechanical strength (tensile strength up to 20 GPa and Young’s modulus approaching 1 TPa).^14^ These remarkable features arise from the sp^2^-hybridized carbon structure, high aspect ratio (length/diameter), and large specific surface area.

These remarkable properties are attributed not only to their hexagonal sp^*2*^-hybridized carbon atoms lattice rolled up in a cylindrical shape but also to their high aspect ratio, α = length (*L*)/diameter (*D*), and small diameter of a few nanometers, leading to a large surface area per unit volume.^15–17^ These cylindrical carbon nanomaterials exhibit characteristics that make them highly promising for several nanotechnology applications. Due to their exceptional stiffness, strength, and toughness, CNTs have been widely explored for applications ranging from nanocomposites to conductive coatings.^18,19^

One of the promising macroscale forms of CNT assembly is buckypaper (BP)—a thin, flexible, porous film composed of entangled CNT networks held together primarily by van der Waals forces.^20,21^ BP is typically fabricated via vacuum-assisted filtration of CNT suspensions, resulting in a highly porous membrane with pore sizes ranging from 10–15 nm, low density, and excellent electrical and thermal conductivities.^22^ These characteristics make BP suitable for diverse applications, including hydrogen storage,^23^ superconductors,^24^ sensors,^25^ actuators,^26^ artificial muscles,^27^ and electrodes in lithium-ion batteries.^28^ BP can be produced using single-walled (SWCNTs),^29^ double-walled (DWCNTs),^30^ and multiwalled carbon nanotubes (MWCNTs).^31^ Production methods vary, including chemical vapor deposition (CVD),^32^ layer-by-layer deposition,^33^ and filtration,^34,35^ with filtration being the most common. In the filtration process, a suspension of CNTs in a liquid medium is filtered through a membrane, separating the CNTs from the solvent and forming the BP.^36^ During this process, individual CNTs agglomerate due to van der Waals forces, forming bundles (ropes) composed of several tens of nanotubes. These bundles create the random porous network characteristic of BP.^37–44^

Several studies have reported the influence of processing parameters on the BP morphology and quality. Smajda et al.^45^ demonstrated that the use of different solvents and CNT concentrations had a limited effect on pore structures. Conversely, Yeh^46^ highlighted that dispersion parameters—such as sonication time, surfactant type, and suspension concentration—strongly influenced the repeatability and uniformity of BPs. In particular, the choice of surfactant (e.g., Triton X-100) plays a key role in promoting homogeneous CNT dispersion and reducing variability.^46^ Zdenko et al.^47^ showed that oxidation treatments could alter the pore structure of MWCNT BPs, while other researchers have emphasized the importance of sacrificial mats and CNT dimensions (length and diameter) in controlling BP morphology.^46,47^ These findings suggest that both processing aids and nanotube geometry must be carefully tuned to produce high-quality BPs. Since BP is essentially the macroscopic result of the assembly of CNTs, its properties are inherently influenced by the geometrical characteristics of the nanotubes. In particular, the aspect ratio (α = *L*/*D*) of MWCNTs plays a crucial role in determining the degree of entanglement, agglomeration, and network formation during filtration. High-aspect ratio nanotubes may enhance interconnectivity but also tend to agglomerate more severely, potentially hindering BP formation. Carbon nanotubes with a lower aspect ratio may facilitate the production of buckypapers with improved uniformity and better dispersion during processing; however, this morphology can also lead to reduced interconnectivity between nanotubes, thereby compromising key functional properties such as the electrical conductivity and mechanical strength of the final structure.

In this context, the present work aims to investigate the influence of the MWCNT aspect ratio and the use of sacrificial mats on the formation, structure, and functional properties of buckypapers. Two commercial grades of MWCNTs were selected: short-type (S-MWCNT, AR ≈ 158) and long-type (L-MWCNT, AR ≈ 600). BPs were prepared by vacuum filtration and characterized by using X-ray diffraction (XRD), Fourier transform infrared spectroscopy (FT-IR), Raman spectroscopy, scanning electron microscopy (FEG-SEM), impedance spectroscopy (IS), electromagnetic interference shielding effectiveness (EMI SE), nitrogen adsorption–desorption (BET), and thermogravimetric analysis (TGA). The results provide key insights into how nanotube geometry and the processing strategy affect the performance of MWCNT-based buckypapers.

## 2 Experimental Section

### 2.1 Materials

Morphological characteristics of MWCNTs are presented in Table 1. Two grades of MWCNTs synthesized via chemical vapor deposition (CVD) with different aspect ratios were chosen:

(1) L-MWCNT: long multiwalled carbon nanotubes supplied by NanoView Nanotechnology (Brazil).
(2) S-MWCNT: shorter multiwalled carbon nanotubes NC7000, supplied by Nanocyl S.A. (Belgium).

**Table 1:** Morphological Characteristics of MWCNTs

morphological characteristics	L-MWCNT	S-MWCNT (NC7000)
Average length (μm)	12	1.5
Average diameter (nm)	20	9.5
Aspect ratio	600	158
Specific surface area (m^2^/g)	150	250–300
Purity (%)	93	90

The nonionic surfactant Triton X-100 (critical micelle concentration: 0.22–0.24 mM; *M*_w_ = 646.87 g/mol) was provided by Dinâmica Qumica Contemporânea Ltd. (Brazil) and was used to disperse the MWCNTs. Acetone (99.6% purity, Dinâmica Qumica) was used to remove residual surfactant.

Polyacrylonitrile (PAN) mats were used as sacrificial materials to assist with BP formation when necessary. PAN (*M_n_* = 267,000 g/mol) was supplied by Radici Group (Brazil). *N*,*N*-dimethylformamide (DMF, 99.6% purity) and isopropyl alcohol (99.8% purity) were also obtained from Dinâmica Qumica and used for PAN removal.

### 2.2 Preparation of PAN Electrospun Nanofibers (PEN) Used as Sacrificial Materials

The preparation of PAN eletrospun nanofibers (PEN) mats was carried out according to Oliveira Junior et al.^48^ The process involved initially dissolving 1.5 g of PAN in 13.5 mL of DMF on a magnetic stirring plate at 120 rpm for 1 h at 100 °C. Subsequently, the solution was placed in an ultrasonic bath for 30 min to obtain a homogeneous mixture. The solution was then transferred to a glass syringe (20 mL) with a 30 mm × 0.8 mm needle. The parameters used in the electrospinning process were: temperature of (25 ± 5) °C, relative humidity of (55 ± 5)%, infusion rate of 1.5 mL/h, working distance (syringe tip to collector) 80 mm, collector rotation speed of 1000 rpm, and voltage 14 kV.^48,49^

### 2.3 Preparation of Buckypapers (BP)

S-MWCNT and L-MWCNT BPs were prepared using the following procedure: 0.2 g of MWCNTs were dispersed in 200 mL of distilled water with the addition of 2 g of surfactant (Triton–X-100), using a high-power sonication (ultrasound tip–Sonics, Vibra-Cell VCX750, model 20 kHz, 750 W). During the dispersion process, the suspension was kept in an ice bath to prevent heating, ensuring that the final temperature did not exceed 25 °C. The ultrasound parameters were 40 min at 40% amplitude, with a pulse system set to on (5 s) and off (3 s). The obtained suspension was then centrifuged at 4000 rpm for 30 min to separate agglomerates from dispersed particles. S-MWCNT BPs were prepared with and without sacrificial mats. L-MWCNT BP, however, requires the use of sacrificial mats. The supernatant was vacuum filtered using a nylon membrane (45 mm diameter, 0.45 μm pore size), with the addition of PAN mats measuring 45 mm × 45 mm. Surfactant removal from the MWCNT network was carried out by using acetone. The BP was carefully removed from the filtration device and dried for 15 h at room temperature.

After this step, the PAN mats were removed from the BPs. Each BP/PEN side was immersed in 25 mL of DMF at 60 °C (Corning, model PC-420D) for 10 min. It was then immersed in 25 mL of isopropyl alcohol for 10 min and dried for 15 h at room temperature.

### 2.4 Characterization of the MWCNT

Both types of MWCNTs were characterized as received by X-ray diffraction (XRD), Raman spectroscopy, and morphological characterization using field emission gun scanning electron microscopy (FEG-SEM).

### 2.5 Characterization of Buckypapers

#### 2.5.1 FEG-SEM

The morphologies of both MWCNTs and BPs were analyzed using a TESCAN MIRA3 FEG-SEM. Sample preparation was performed using carbon double-sided tape, followed by coating with a thin layer of gold via a sputtering system. The samples were analyzed at an operating voltage of 5 kV under high vacuum, with an In Beam SE (secondary electron) detector and a working distance of 4 mm.

#### 2.5.2 Nitrogen Gas Adsorption at 77 K

Textural properties, surface area, and total pore volume of BPs were evaluated using Quantachrome Nova-e series equipment. Gas adsorption–desorption isotherms were obtained with relative pressure *P*/*P*_0_ ranging from 0.05 to 0.98. Sample preparation involved drying in a vacuum oven for 15 h at 80 °C, followed by degassing for 3 h at 80 °C under vacuum. Surface area was measured by the Brunauer–Emmett–Teller (BET) method in a *P*/*P*_0_ range of 0.05 to 0.35, and total pore volume was calculated at a relative pressure of *P*/*P*_0_ = 0.98. All data were analyzed by using NovaWin 11.01 software.

#### 2.5.3 X-ray Diffraction (XRD)

X-ray diffraction patterns of both MWCNTs were analyzed to determine their diameters and the number of walls. X-ray diffraction patterns of both MWCNTs and BPs measurements were conducted using a Rigaku diffractometer (Ultima IV model) operating at 40 kV and 30 mA, with copper Kα radiation (λ = 1.54056 Å) and a Nickel filter to block Kβ radiation. A 2θ range of 10°–60° was scanned at a rate of 10° per minute. The average thickness of MWCNTs was estimated using Scherrer’s equation (eq 1), while the average number of walls was determined as a ratio of the average thickness to the intertube distance, calculated using Bragg’s law (eq 2).^50,51^

1 $$\tau =\frac{K\times \lambda }{\beta \times \cos\theta }$$

where τ is the mean size of the ordered (crystalline) domains; *K* is a dimensionless shape factor (0.9); λ is the X-ray wavelength; β is the line broadening at half the maximum intensity; and θ is the Bragg angle.

2 nλ=2d⁡sin⁡θ

where λ is the wavelength, θ is the glancing angle, and *d* is a grating constant.

#### 2.5.4 Raman Spectroscopy

Raman spectroscopy analyses of MWCNTs were conducted to assess their structural integrity. Raman scattering spectroscopy of BPs and both MWCNTs was performed using Horiba LabRAM HR Evolution equipment with a 532 nm laser radiation source and a laser spot diameter of 1.54 μm. Spectra were collected with three accumulations of 30 s each in the 500 to 3000 cm^–1^ range.

#### 2.5.5 Fourier Transform Infrared Spectroscopy (FT-IR)

FT-IR spectroscopy was performed by using a PerkinElmer Spectrum 2000 spectrophotometer. Scanning was conducted in the 4000 to 600 cm^–1^ range with a resolution of 4 cm^–1^ after 20 scans.

#### 2.5.6 Thermogravimetric Analysis (TGA)

Thermogravimetric analysis (TGA) was performed on Netzsch Iris TG F1 equipment with a heating rate of 20 °C/min under nitrogen flow and synthetic air at 50 mL/min, within a temperature range from 40 to 800 °C. The mass of samples was 0.01 g.

#### 2.5.7 Impedance Spectroscopy

Electrical characterization was performed through impedance spectroscopy (IS) measurements using an alternating current impedance spectrometer, a Solartron SI 1260 Impedance/Gain-phase Analyzer. A voltage of 0.5 V was applied at frequencies ranging from 1 to 10^6^ Hz, capturing 50 points for each BP, and the contact area was 1.33 cm^2^. The sample thickness (*l* = 0.062 to 0.117 ± 0.025 mm) was measured by a digital micrometer. A thin layer of gold/palladium alloy was deposited on both sides of the samples by using a metallizer (Bal-tec, MED020) to form the electrical contact, creating a metal-composite-metal structure. Three samples of each composition were analyzed. The AC electrical conductivity (σ_AC_) of the materials was calculated based on the sample thickness (*l*), contact area (*A*), and impedance modulus (|Z|) values obtained from the analysis, as shown in eq 3.

3 $$\sigma_{\mathrm{AC}}=\frac{l}{|Z|\times A}$$

#### 2.5.8 Electromagnetic Interference Shielding Effectiveness (EMI SE)

A vector network analyzer (VNA, Agilent Technologies, model PNA-L N5235A) with a coupled waveguide (W*R*-90) was operated to obtain the complex scattering transmission parameter (*S*_21_) of the buckpapers in the X-band (8.2–12.4 GHz). The complex scattering parameters *S*_12_ (correlated to the transmittance [T]) and *S*_11_ (correlated to the reflectivity [R]) obtained were applied to calculate the values of the total attenuation effectiveness (SE_T_). SE_T_ can be defined in terms of a reflective and absorption attenuation effectiveness (SE_R_ and SE_A_, respectively), according to the (eqs 4–7).^11^ Three samples of each composition were analyzed.

4 $${\mathrm{SE}}_{T}\left(\mathrm{dB}\right)=10\log_{10}\left(\frac{1}{\left({\left|S_{21\left(\mathrm{mag}\right)}\right|}^{2}\right)}\right)=10\log_{10}\left(\frac{1}{T}\right)$$

5 $${\mathrm{SE}}_{R}\left(\mathrm{dB}\right)=10\log_{10}\left(\frac{1}{{1-\left|S_{11\left(\mathrm{mag}\right)}\right|}^{2}}\right)=10\log_{10}\left(\frac{1}{1-R}\right)$$

6 $${\mathrm{SE}}_{A}\left(\mathrm{dB}\right)=10\log_{10}\left(\frac{{1-\left|S_{11\left(\mathrm{mag}\right)}\right|}^{2}}{\left({\left|S_{21\left(\mathrm{mag}\right)}\right|}^{2}\right)}\right)=10\log_{10}\left(\frac{1-R}{T}\right)$$

7 A+R+T=1

where *S*_21(mag)_ is the *S*_21_ in magnitude, *T* is *S*_21(mag)_^2^, *S*_11(mag)_ is the *S*_11_ in magnitude, and *R* is *S*_11(mag)_^2^.

## 3 Results and Discussion

### 3.1 Characterization of the MWCNTs

Figure 1 A presents the X-ray diffraction (XRD) patterns for the S-MWCNT and L-MWCNT, showing similar diffraction profiles. The most intense peak for S-MWCNT appears at approximately 25.7°, while for L-MWCNT, it is slightly shifted to 25.8°, both corresponding to the (002) diffraction plane.^52^ Additional lower-intensity peaks were observed in both samples (around 43°–45°), likely attributed to a combination of the (100) and (101) diffraction planes.^52^ Based on these values, the interlayer distance and average diameter were estimated as 3.47 Å and 9.5 nm for S-MWCNT, and 3.45 Å and 20 nm for L-MWCNT, respectively. The ratio of these parameters allowed estimation of the number of walls, approximately 8 for the S-MWCNT and 13 for the L-MWCNT.

**Figure 1:**
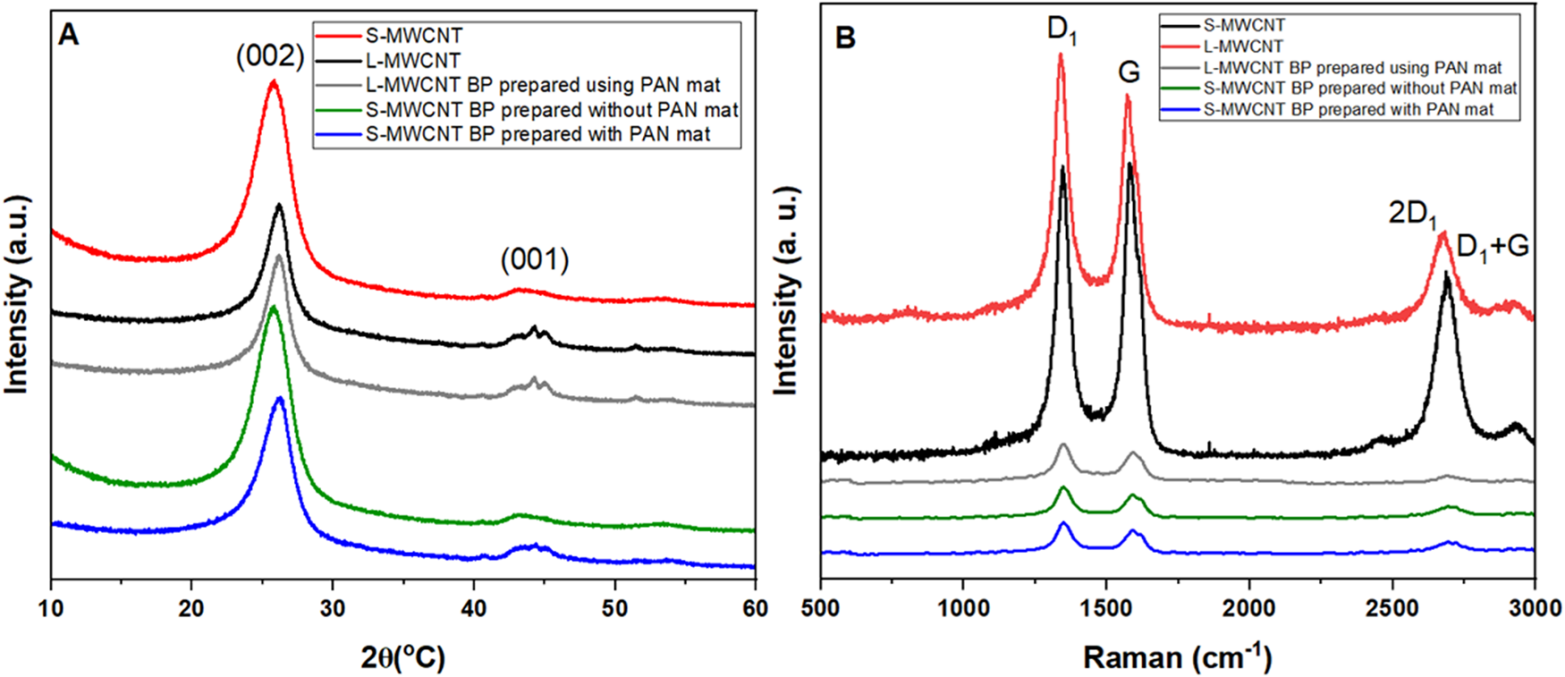
(A) X-ray diffraction patterns with 2θ from 10 to 60 (°C) and (B) Raman spectra from 500 to 3000 cm^–1^ of S-MWCNT and L-MWCNT as received and S-MWCNTs BPs prepared with and without sacrificial PAN mat, and L-MWCNTs BP prepared with sacrificial PAN mat in the filtration process.

As expected, the L-MWCNT sample exhibits a slightly more intense and narrower peak around 25.8°, indicating a larger diameter and greater number of walls, in agreement with the supplier’s specifications.

Figure 1 B presents the Raman spectra for the S-MWCNT and L-MWCNT as received. The spectra exhibit characteristic D and G bands, typical of sp^*2*^ hybridized carbon-based materials. The D band, located between 1340–1341 cm^–1^, is associated with sp^*3*^-hybridized carbon atoms and indicates structural defects in the MWCNTs, such as vacancies and edges. In contrast, the G band, observed between 1572–1575 cm^–1^, corresponds to the E_2g_-type vibrational modes in the axial parallel planes of the MWCNTs and is attributed to sp^*2*^-hybridized carbon atoms, reflecting the structural integrity of the nanotubes.^53,54^

Additionally, the Raman spectrum revealed the 2D band, located around 2676–2682 cm^–1^, which is associated with second-order D-band interactions and double resonance phenomena. Notably, the L-MWCNTs exhibit a higher 2D band intensity, indicating greater structural organization.^55^ The intensity ratio between the D and G bands (*I*_D_/*I*_G_) is an important parameter for assessing the structural integrity of the MWCNTs, the greater ratios being associated with a higher defect concentration. The *I*_D_/*I*_G_ of S-MWCNT is 1.01, and that of L-MWCNT is 1.15. Furthermore, this ratio enables the estimation of the average defect distance (*L*_a_) in the nanotube structure (*L*_a_ of S-MWCNT is 16.8 nm and L-MWCNT is 17.1 nm).^7,53,54^

Figure 2 presents FEG-SEM images of both MWCNTs. As expected, the L-MWCNTs exhibit a greater length and larger diameter (Table 1). Additionally, small CNTs are observed among the L-MWCNTs, suggesting a broader size distribution. These nanotubes also tend to form larger and denser clusters (Figure 2A). In contrast, the S-MWCNT displays a shorter length and smaller diameter with a more homogeneous distribution. Although the S-MWCNT also agglomerates into clusters (Figure 2B), these appear smaller and less dense than those observed for L-MWCNT. These morphological characteristics align with findings in the literature.^7,56^ All of these observations are closely related to the BP formation and characterization results, which are discussed in the following sections.

**Figure 2:**
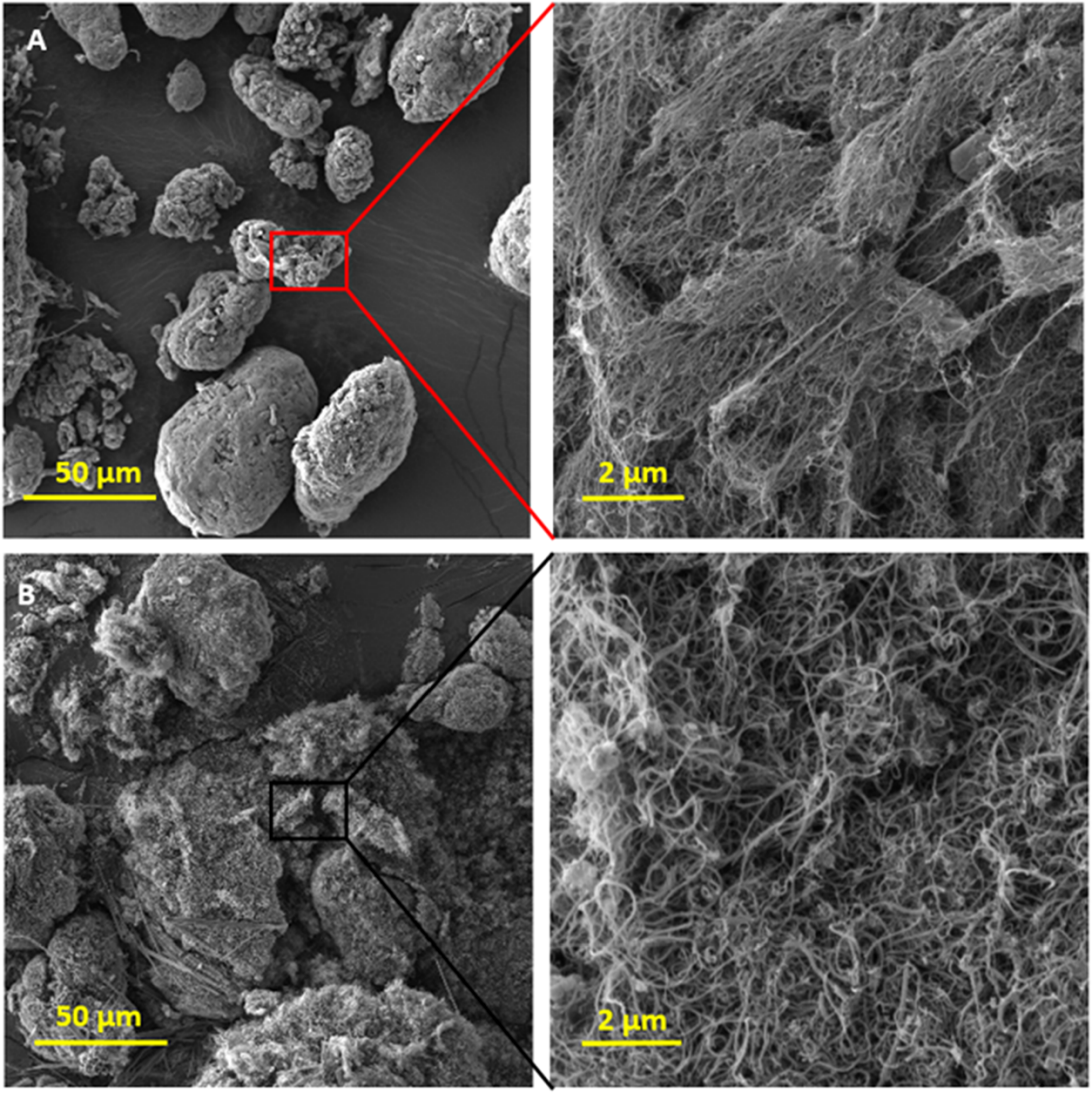
FEG-SEM images of (A) L-MWCNTs and (B) S-MWCNTs with different magnifications of 500× and 10,000×.

### 3.2 Characterization of the BPs

Figure 3 displays macroscopic and FEG-SEM images of the buckypapers (BPs) fabricated with the L-MWCNT and S-MWCNT, both with and without the use of sacrificial PAN mats.

**Figure 3:**
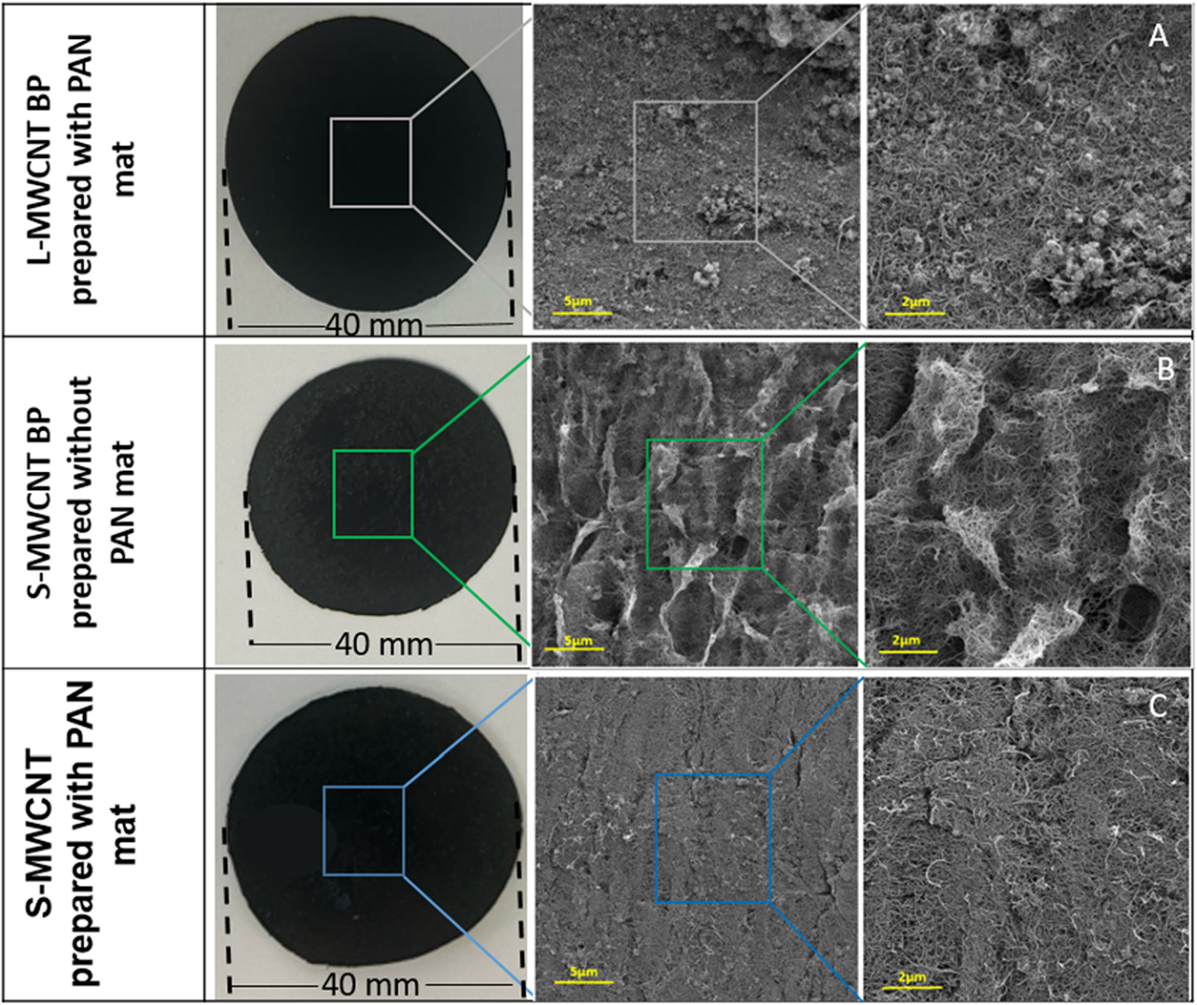
Macroscopic images and FEG-SEM images of (A) L-MWCNT BP, (B) S-MWCNT BP, and (C) S-MWCNT BP prepared using sacrificial material during the filtration process with different magnifications of 10,000× and 25,000×.

The buckypaper (BP) produced with L-MWCNTs (Figure 3A) exhibited a thickness of 0.117 mm, which was greater than that of the BPs fabricated with S-MWCNTs, measuring 0.062 mm without the PAN mat (Figure 3B) and 0.095 mm when the PAN mat was included (Figure 3C). Despite the greater thickness, the L-MWCNT-based BP appeared slightly more brittle, likely due to the larger size of the nanotubes and their stronger tendency to agglomerate. This behavior may be associated not only with the greater length and diameter of the L-MWCNTs relative to the S-MWCNTs but also with the fact that, for a given mass, nanotubes with larger diameters are fewer in number. This reduction in the CNT population, together with their higher aspect ratio, can significantly affect the packing density and entanglement of the network, thereby influencing both the morphology and the mechanical performance of the produced buckypaper. The longer length promotes more effective entanglement between the nanotubes, while the smaller diameter increases the specific surface area, enhancing van der Waals interactions. Consequently, nanotubes with reduced dimensions provide a larger contact area per unit mass, which can strongly affect the morphology and mechanical performance of the buckypaper. These characteristics contribute to a greater tendency for the formation of larger agglomerates and bundles, hindering the homogeneous dispersion in the material. The buckypaper obtained from L-MWCNTs exhibited greater thickness but lower structural uniformity compared with the thinner and more homogeneous films formed by S-MWCNTs. The observed differences in morphology, combined with variations in nanotube entanglement, packing density, and intrinsic mechanical quality of the CNTs themselves (often reduced in larger-diameter tubes due to structural imperfections), are expected to influence the overall mechanical performance of the buckypaper. As observed in the FEG-SEM images of the raw materials (Figure 2), the L-MWCNTs exhibit greater length and diameter, which promotes the formation of larger aggregates. Conversely, the S-MWCNT-based BP exhibited a more uniform morphology with stronger interactions between nanotubes, leading to a more homogeneous and mechanically stable structure.

The higher aspect ratio of L-MWCNTs increases the stability of their agglomerates, resulting in lower porosity and making it more difficult to form a thin film. The addition of a sacrificial material improves the porosity of the BP. In contrast, S-MWCNTs have smaller dimensions, which creates more pores between the CNTs and facilitates BP formation.

Figure 3 presents images of the BP samples and their corresponding FEG-SEM micrographs. BP production was successfully achieved using both types of MWCNTs; however, obtaining BP with L-MWCNTs proved to be more challenging, requiring the use of a sacrificial material. This limitation probably stems from the stability of their agglomerates, due to their higher aspect ratio, tending to agglomerate among themselves rather than interacting with neighboring nanotubes, preventing the formation of the continuous network necessary for BP stability. Without a sacrificial material, BP formation was hindered or entirely impossible.

In contrast, BP formation with S-MWCNTs was possible without the addition of sacrificial materials. However, for comparison purposes, S-MWCNT BPs were prepared both with (Figure 3C) and without (Figure 3B) a sacrificial PAN mat. In Figure 3A–C, the presence of agglomerates is evident, likely due to the influence of the sacrificial PAN mat, whereas in Figure 3B, a more homogeneous surface and the presence of entangled MWCNTs can be observed.

Thus, the use of sacrificial PAN mats had a noticeable effect on the BP morphology. In BPs fabricated with PAN mats (Figure 3A–C), residual agglomerates and less uniform surfaces were observed. The sample without mat (Figure 3B) displayed a smoother and more continuous CNT network, suggesting improved dispersion and connectivity.

Quantitative image analysis was performed using ImageJ and Python-based routines. Surface porosity was calculated by Otsu thresholding, identifying dark regions as voids. The results are summarized in Table 2. Bundle diameter distribution and agglomerate area fraction were obtained through segmentation and skeleton-based measurements. The quantitative image analysis reveals clear morphological differences among the three buckypapers. The L-MWCNT BP prepared with the PAN mat shows extremely low surface porosity (0.89%), indicating a highly compact and well-entangled network typical of long nanotubes. In contrast, both S-MWCNT BPs exhibit very high porosities (>85%), reflecting a much more open and loosely connected structure.

**Table 2:** Quantitative Image Analysis: Surface Porosity, Bundle Diameter Distribution, and Agglomerate Area Fraction of BPs

sample	surface porosity (%)	agglomerate area fraction	mean bundle diameter (μm)
L-MWCNT BP prepared with PAN mat	0.89	0.121 ± 0.019	0.85 ± 0.27
S-MWCNT prepared with PAN mat	88.5	0.125 ± 0.030	1.06 ± 0.39
S-MWCNT BP prepared without PAN mat	85.6	0.149 ± 0.018	0.38 ± 0.25

Agglomerate area fraction increases from L-MWCNT (0.121) to S-MWCNT with a PAN mat (0.125) and reaches its highest value in the S-MWCNT BP without a PAN mat (0.149), suggesting poorer dispersion when the mat is absent. The mean bundle diameter follows a complementary trend: S-MWCNT with a PAN mat forms the thickest bundles (1.06 μm), whereas the sample without a PAN mat shows thinner bundles (0.38 μm) but greater agglomeration. The L-MWCNT BP exhibits an intermediate bundle size consistent with its dense microstructure.

Overall, these results indicate that nanotube length predominantly governs network packing and homogeneity, while the PAN mat provides secondary benefits by reducing agglomeration and stabilizing the bundle structure, especially for short nanotubes.

Figure 4 shows the N_2_ adsorption/desorption isotherms for the BP samples. The isotherms do not strictly conform to a well-defined IUPAC classification but generally resemble a Type IV isotherm, which is characteristic of mesoporous materials and associated with pore condensation.^57–59^ The BP exhibits an H1-type hysteresis loop, indicative of materials with well-defined cylindrical pores and significant capillary condensation of N_2_ gas within the internal cavities.^59^

**Figure 4:**
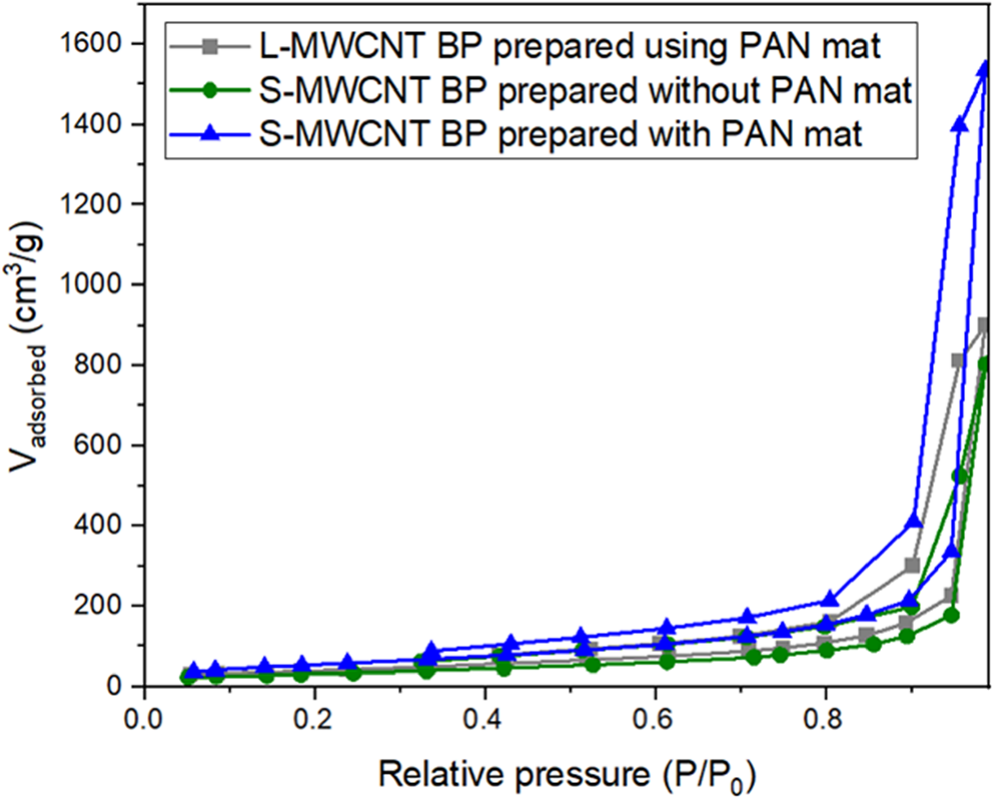
N_2_ adsorption and desorption isotherms with relative pressure from 0.05 to 0.98 (*P*/*P*_0_) of S-MWCNTs BPs prepared with and without a sacrificial PAN mat, and L-MWCNTs BP prepared with a sacrificial PAN mat in the filtration process.

Table 3 presents the surface area and total pore volume of the BP samples. The L-MWCNT prepared using the sacrificial PAN mat exhibits a lower total pore volume (0.349 *V*_0_._95_) compared to that of S-MWCNT BP prepared without the sacrificial mat (0.520 *V*_0_._95_), though it remains higher than that of S-MWCNT BP prepared with the sacrificial PAN mat (0.275 *V*_0_._95_). This suggests that a portion of the PAN polymer chains may be infiltrating the MWCNT or sealing their edges, thereby obstructing the nanotubes, as reported by other authors.^51,60,61^

**Table 3:** Surface Area (*S*_BET_) and Total Pore Volume (*V*_0_._95_) Values for S-MWCNTs BPs Prepared with and without a Sacrificial PAN Mat and L-MWCNTs BP Prepared with a Sacrificial PAN Mat in the Filtration Process

samples	*S*_BET_ (m^2^/g)	*V*_0.95_ (cm^3^/g)
L-MWCNT BP prepared with PAN mat	145	0.349
S-MWCNT BP prepared without PAN mat	205	0.520
S-MWCNT prepared with PAN mat	116	0.275

In addition, the use of sacrificial mats decreased the surface area of the BPs. The surface area calculated using the BET method for L-MWCNT BP prepared with a sacrificial mat was 145 m^2^/g, while for S-MWCNT BP prepared without the mat, it was 205 m^2^/g, and for that prepared with the mat, it was 116 m^2^/g. With a larger surface area, the S-MWCNT exhibited greater interaction with each other, resulting in less brittle and more flexible BP, as demonstrated by their morphological characteristics. Furthermore, the surface area values are similar to those reported in other studies.^59–61^

Figure 1 A shows the X-ray diffraction (XRD) patterns of S-MWCNT BPs prepared with and without a sacrificial PAN mat as well as L-MWCNT BP prepared using a sacrificial PAN mat. As observed, there is no significant difference between the XRD patterns of the BPs compared to the neat materials. The diffraction peaks at 25.8° (002) and 44° (100), corresponding to the graphitic structure of MWCNTs, are visible.^61,62^

Figure 1 B presents the Raman spectra of BP samples from both MWCNT types. In this study, the first-order peaks are designated as D_1_ and G, and the second-order peaks are identified as 2D_1_ and D_1_ + G, whose positions and origins are discussed below.^63,64^ No significant differences were observed in the Raman spectra of BP samples from different carbon nanotubes. Both spectra exhibited characteristic peaks of carbon nanotubes, consistent with findings in the literature.^67^

The first-order Raman spectra exhibit two prominent peaks: G (ωG = 1405 cm^–1^) and D_1_ (ωD_1_ = 1375 cm^–1^). The G peak corresponds to the C=C stretching vibration mode in aromatic rings of graphitic structures or olefinic chains.^68^ The D_1_ peak, known as the disorder mode, is defect-activated and corresponds to the A1-type mode at the K point of the Brillouin zone.^68^

The *I*_D_/*I*_G_ ratio determined by Raman spectroscopy was used as an indicator of defect density in the nanotubes and buckypapers (BP). The L-MWCNTs exhibited an average *I*_D_/*I*_G_ value of 1.01, whereas the S-MWCNTs showed a higher value of 1.15, indicating a greater defect density in the shorter CNTs. After buckypaper fabrication, the *I*_D_/*I*_G_ values became very similar (1.018 for the L-MWCNT BP prepared with a PAN mat, 1.017 for the S-MWCNT BP prepared without a PAN mat, and 1.019 for the S-MWCNT BP prepared with a PAN mat), suggesting that, at the BP level, there is no significant difference in the *I*_D_/*I*_G_ ratio among the three conditions.

Figure 5 shows the FT-IR spectra of buckypapers (BP) prepared from S-MWCNT and L-MWCNT with and without a sacrificial PAN mat. Overall, the spectra are very similar, which is expected for largely graphitic and highly absorbing carbon materials. The most notable differences are the characteristic PAN bands (C≡N at ∼2242 cm^–1^, C=N at ∼1662 cm^–1^, and CH_2_/CH_3_ at ∼1451 cm^–1^), which appear only in samples prepared using the sacrificial PAN mat, indicating residual polymer. Weak features near 2900 and 970 cm^–1^ are tentatively attributed to C–H vibrations and out-of-plane modes; however, these bands are close to the noise level and should be interpreted with caution. Given the inherently low FT-IR sensitivity for black carbonaceous samples, we rely mainly on the presence/absence and relative intensity of the PAN-related bands rather than on minor spectral variations between CNT types.^65,66^ The limited contrast in FT-IR among the different BP samples is attributed to the strong light absorption and weak IR activity of graphitic carbon; therefore, conclusions regarding surface chemistry are supported by complementary analyses (Raman, TGA, and SEM) rather than FT-IR alone.

**Figure 5:**
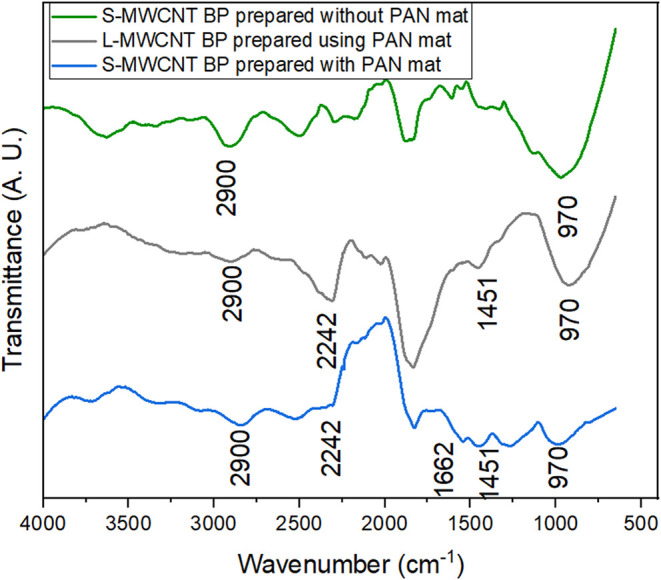
FT-IR spectra with wavenumber from 4000 to 600 cm^–1^ of S-MWCNTs BPs prepared with and without sacrificial PAN mat, and L-MWCNTs BP prepared with sacrificial PAN mat in the filtration process.

Figure 6 presents the TGA and DTG curves for the BPs under both oxidizing (synthetic air) and inert (nitrogen) atmospheres. Table 4 lists the initial degradation temperatures (*T*_initial_), the temperature of the maximum decomposition rate (*T*_max1_ and *T*_max2_, corresponding to the two more pronounced minima of the derivative curve), and the residual mass for all BPs obtained under an oxidizing atmosphere.

**Figure 6:**
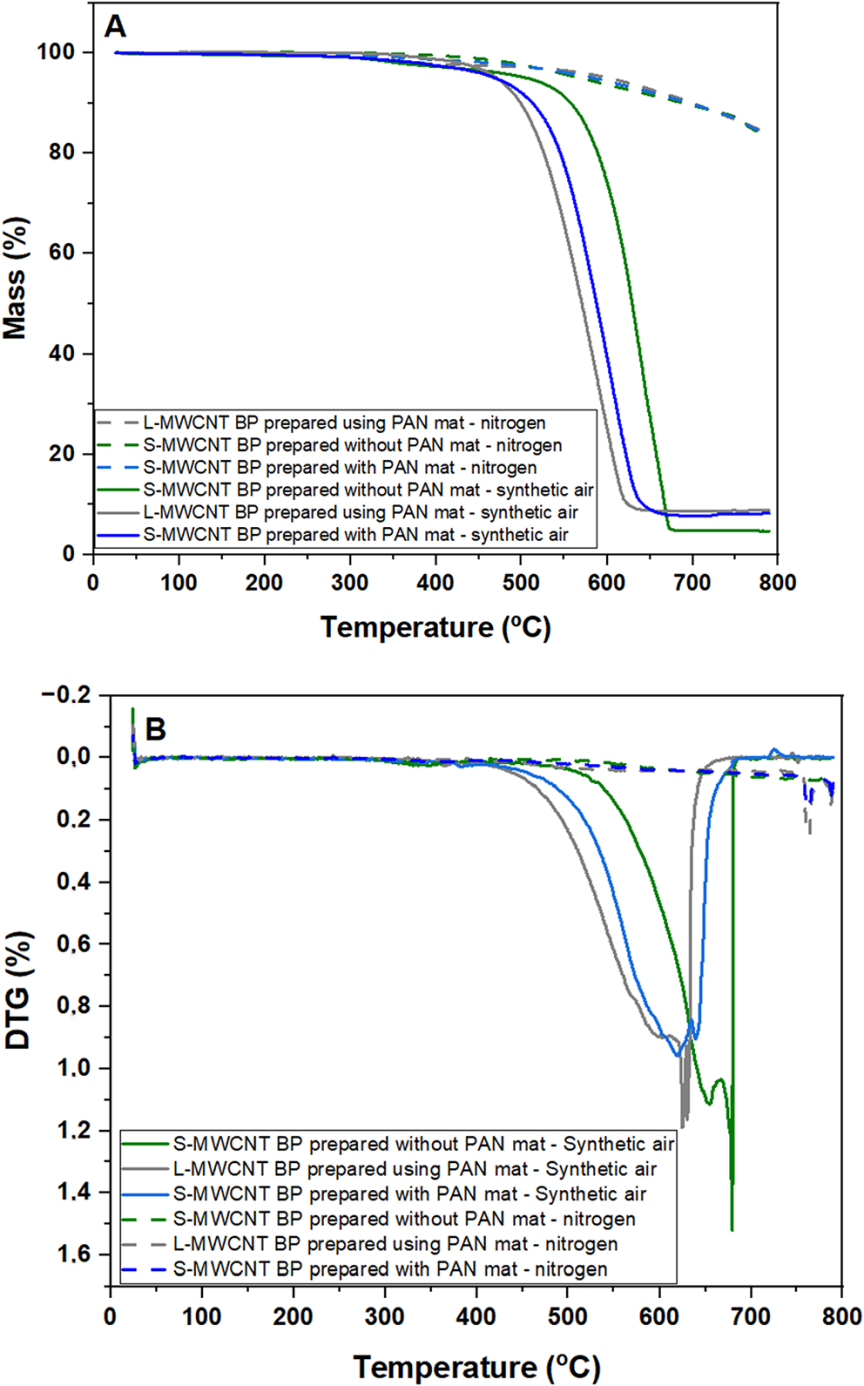
(A) TGA and (B) DTG curves with temperatures from 40 to 800 °C of S-MWCNTs BPs prepared with and without a sacrificial PAN mat, and L-MWCNTs BP prepared with a sacrificial PAN mat in the filtration process.

**Table 4:** Thermal Degradation Initial Temperature (*T*_initial_), Maximum Decomposition Rate Temperatures (*T*_max1_ and *T*_max2_), and Residual Mass for BPs Obtained in an Oxidizing Atmosphere

samples	*T*_initial_ (°C)	*T*_max 1_ (°C)	*T*_max 2_ (°C)	residual mass at 800 °C (%)
S-MWCNT BP prepared without PAN mat	380	656	680	8.7
L-MWCNT BP prepared with PAN mat	442	625	630	4.8
S-MWCNT BP prepared with PAN mat	311	619	640	8.1

Under an oxidizing atmosphere, carbon undergoes oxidation^69–71^ at temperatures above 500 °C, resulting in thermal degradation, thus limiting its applications to lower temperatures.^72,73^ This is particularly important for BP made with L-MWCNT and S-MWCNT in synthetic air, leaving residual masses of approximately 4.8 and 8.0%, respectively. This residual mass can be attributed to catalytic metals and inorganic impurities in the MWCNT.^74^ For the S-MWCNT prepared without a sacrificial PAN mat, in synthetic air, its higher mass loss is observed between approximately 620 and 680 °C, with a total mass loss of about 8.0%, showing higher resistance to the temperature. In both cases, L-MWCNT and S-MWCNT are completely vaporized under the oxidizing atmosphere, forming CO_2_ and CO compounds.^71^

The initial degradation temperature (*T*_initial_) was defined as the onset temperature, obtained from the intersection of the baseline with the tangent drawn at the inflection point of the main weight-loss step. According to this definition, the *T*_initial_ values of the BPs derived from the L-MWCNT and S-MWCNT prepared with a sacrificial PAN mat differ only slightly. It is worth noting, however, that in the case of the S-MWCNT-based BPs, a small mass loss is already observed at lower temperatures, which may be associated with surface species or with residual PAN, rather than with the degradation of the CNT framework itself.

Larger carbon-based nanoparticles with more extensive and structurally perfect hexagonal lattices of sp^2^-hybridized carbon atoms are expected to exhibit higher thermal stability compared to those with more defective structures. This trend is consistent with the subtle difference observed in *T*_initial_ between L-MWCNT- and S-MWCNT-based BPs prepared with a sacrificial PAN mat. Both types of BPs, however, showed significantly lower degradation temperatures than the sample prepared without the sacrificial mat, suggesting that this behavior is at least partly associated with the presence of residual PAN in the BP. Such residues are known to undergo oxidation at relatively low temperatures, which could accelerate the overall degradation process and thus represent a drawback of using sacrificial mats.

Under an inert atmosphere, the TGA analysis of all BPs shows a gradual mass loss up to 800 °C, leaving a residual mass of approximately 85%. The similarity in thermal behavior across all samples in nitrogen reflects the intrinsic high thermal stability of CNTs and also the carbonization of PAN in the absence of oxygen. The observed mass loss under this condition is likely related to less-ordered CNT regions and surface defects, such as functional groups at the edges or outer walls of the nanotubes.

FT-IR spectra show the C≡N stretching band (∼2242 cm^–1^), indicating a partial PAN residue. TGA revealed a small additional mass loss of ∼5 wt % between 300 and 350 °C, attributed to PAN decomposition. These results suggest incomplete removal of PAN after solvent washing. The influence of this minor residue on the electrical measurements is expected to be negligible compared to contact resistance.

Impedance spectroscopy (IS) is a valuable technique for evaluating the electrical behavior of BP and providing insights into its morphology.^75–77^ The AC electrical conductivity behavior, derived from the IS of BP, is shown in Figure 7.

**Figure 7:**
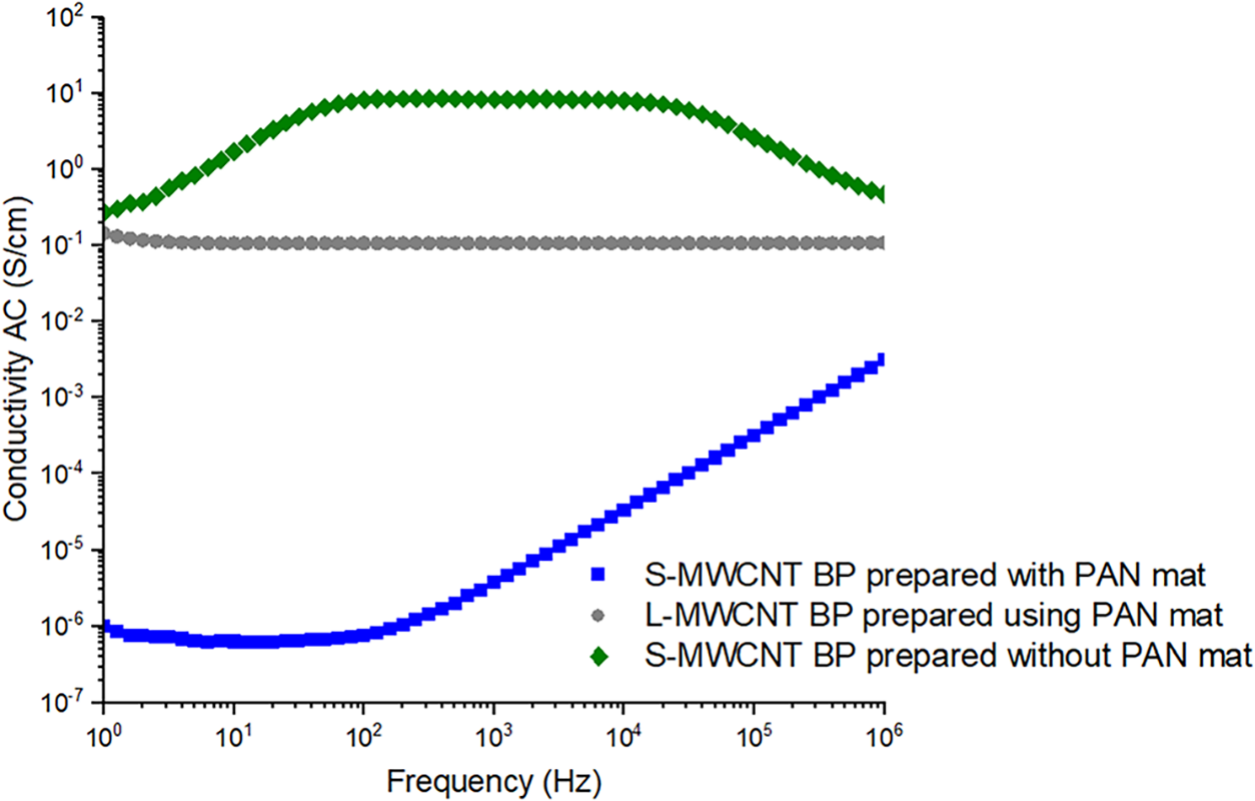
Electrical conductivity AC with frequency from 1 to 10^6^ Hz for S-MWCNTs BPs prepared with and without a sacrificial PAN mat, and L-MWCNTs BP prepared with a sacrificial PAN mat in the filtration process.

It can be observed that the S-MWCNT BP prepared without a sacrificial PAN mat and the L-MWCNT BP prepared with the sacrificial PAN mat exhibit characteristics of an electrically conductive material, with high conductivity values (>10^–1^ S/cm) and being practically frequency-independent.^7^ However, the S-MWCNT BP prepared without a sacrificial mat shows slightly higher electrical conductivity values compared to the L-MWCNT BP. At 1 kHz, the S-MWCNT buckypaper prepared without the PAN mat shows a conductivity of approximately 3 × 10^–1^ S/cm, while the L-MWCNT buckypaper prepared with PAN mat reaches about 1 × 10^1^ S/cm, indicating an order-of-magnitude increase relative to the S-MWCNT sample without PAN mat. In contrast, the S-MWCNT buckypaper prepared with the PAN mat exhibits a much lower conductivity, close to 1 × 10^–5^ S/cm at the same frequency. At higher frequencies (10^5^–10^6^ Hz), the S-MWCNT sample with PAN mat increases to approximately 10^–3^ S/cm, whereas the S-MWCNT sample without PAN remains nearly frequency-independent at ∼3 × 10^–1^ S/cm around 10^2^–10^3^ Hz. These differences can be attributed to processing conditions such as the use of the sacrificial mat, dispersion, and the distribution of MWCNT in BP. On the other hand, the S-MWCNT BP prepared with the sacrificial PAN mat exhibited behavior characteristic of a semiconductor material, likely due to mat residues remaining in the samples, which hinder the passage of electric current. This effect is more pronounced in the S-MWCNT BP, probably because it has a lower thickness, making the impact of the mat thickness more significant, while the thicker and less homogeneous L-MWCNT BP prepared with the sacrificial PAN mat exhibits less pronounced effects.

Overall, the BP made from S-MWCNTs without the use of a sacrificial PAN mat exhibited a more uniform morphology and better interaction between the carbon nanotubes, resulting in a less fragile and more uniform BP, which facilitates the passage of electric current. Therefore, both the use of a sacrificial material and the aspect ratio of the MWCNTs affect the electrical properties of the BPs, and these factors are crucial for the intended application of this BP.

Electromagnetic interference shielding effectiveness (EMI SE) is shown in Figure 8. A commonly accepted criterion for effective shielding is SE_T_ ≥ 20 dB for 2 mm thick samples.^78^ Despite their submicrometric thickness, all BPs exhibited excellent attenuation performance, with L-MWCNT BPs achieving attenuation values of approximately 19–20 dB and S-MWCNT BPs demonstrating significantly higher values, ranging from 33 to 36 dB. The lower attenuation for L-MWCNT BP corroborates the previous results and may be explained by the presence of defects in these BP as agglomerates and/or imperfections.

**Figure 8:**
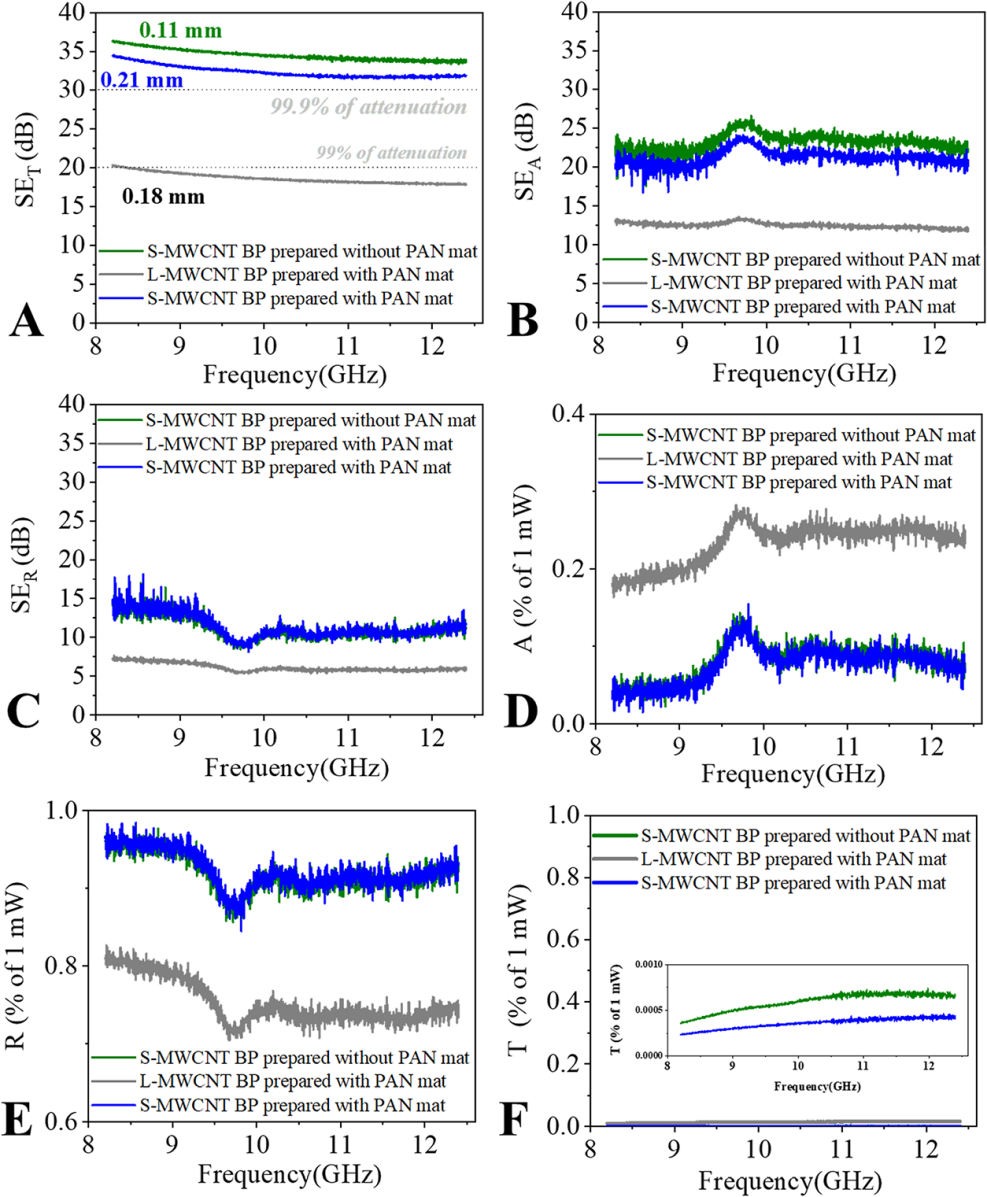
Electromagnetic shielding performance of different types of MWCNT buckypapers (BP) prepared with and without PAN mat in the 8.2 to 12.4 GHz frequency range. (A) Shielding effectiveness by reflection (SE_R_), (B) shielding effectiveness by absorption (SE_A_), (C) total shielding effectiveness (SE_T_), (D) absorption coefficient, (E) reflection coefficient, and (F) transmission coefficient, with the inset showing the expanded low-transmission region.

Unlike the trend observed in electrical conductivity, the presence of insulating PAN layers had a minimal effect on the attenuation behavior. Both S-MWCNT BPs displayed similar attenuation values, which was expected since the electromagnetic field propagates through the sample, and the PAN sacrificial mat layer acts as an electromagnetically transparent medium.

High attenuation behavior in BPs has been previously reported in the literature by Ribeiro et al.,^79,80^ and the enhanced electrical conductivity of MWCNTs contributes to an excellent shielding performance, comparable to that of metals.

Evaluating the shielding components of both S-MWCNT BP with and without PAN mats, higher reflection- and absorption-based attenuations (SE_R_ and SE_A_) were observed compared with the L-MWCNT BPs (Figure 8B,C). This indicates that the L-MWCNT BP exhibits lower surface reflectivity and is less effective in promoting internal electromagnetic loss mechanisms, which in these BPs are likely dominated by ohmic (conduction) losses. The reduced SE_R_ and SE_A_ of the L-MWCNT BPs may be associated with a higher defect content in their structure, corroborating the earlier discussion of processing difficulties arising from their larger aspect ratio.

Comparing the two BP types, the presence of the PAN mat slightly decreased the absorption-based attenuation (SE_A_), particularly in the S-MWCNT + mat sample, suggesting a reduction in certain loss mechanisms due to the more insulating behavior of residual PAN. This effect, however, is more noticeable on a logarithmic scale.

When the power distribution coefficients (*A*, *R*, and *T*) were examined (Figure 8D–F), both S-MWCNT samples exhibited nearly identical behavior. These coefficients further confirm the highly reflective, metal-like shielding behavior of the BPs, characterized by a high reflection coefficient (*R* > 80%) and comparatively lower absorption (*A*), consistent with a reflection-dominated shielding mechanism.

In Figure 9, a graphical diagram differentiates the mechanisms of the three BPs studied here.

**Figure 9:**
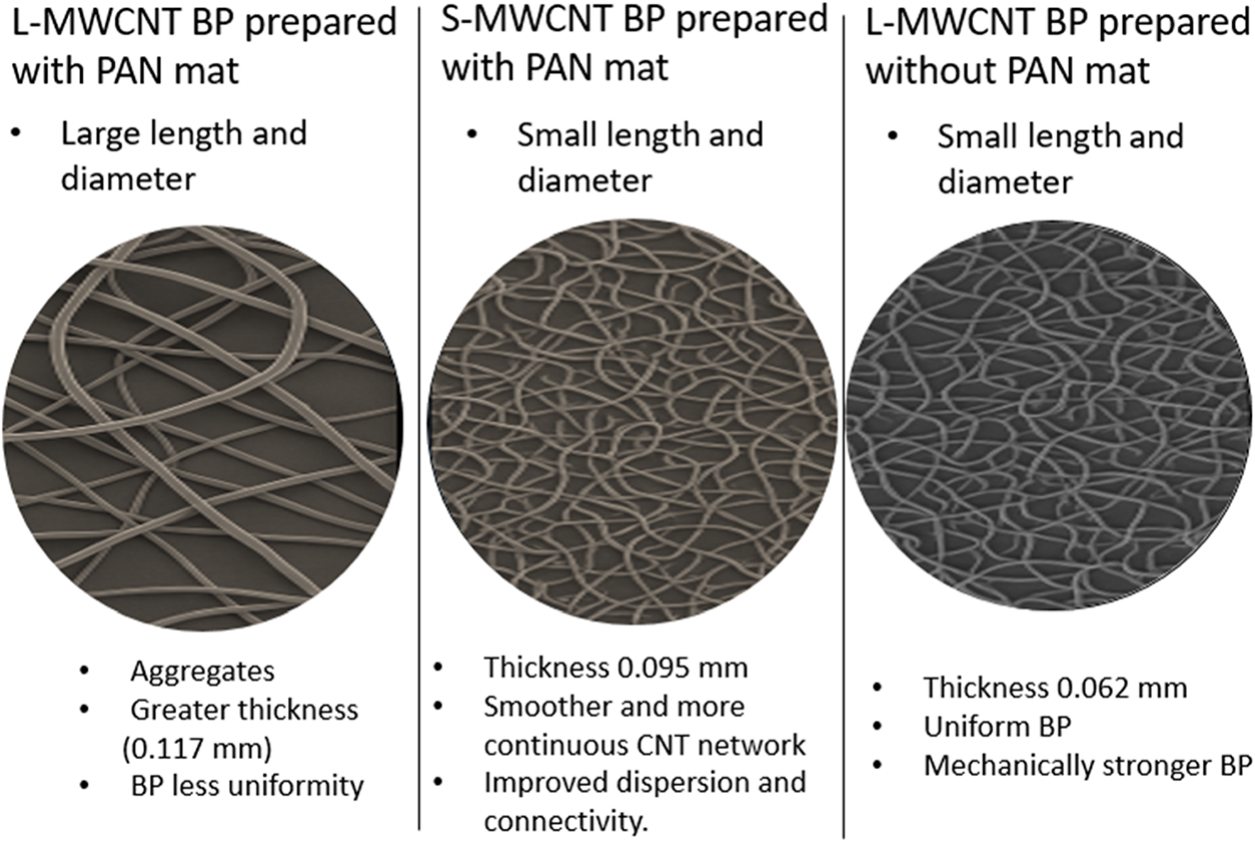
Graphical diagram differentiates the mechanisms of the three BPs studied here.

## 4 Conclusions

Buckypapers (BPs) were successfully fabricated using multiwalled carbon nanotubes (MWCNTs) with different aspect ratios (AR ≈ 158 and AR ≈ 600) via vacuum-assisted filtration. The results demonstrate that both the aspect ratio of the MWCNTs and the use of sacrificial polyacrylonitrile (PAN) mats play critical roles in determining the structural, electrical, and electromagnetic properties of the resulting BPs.

BPs prepared with high-aspect-ratio L-MWCNTs (AR ≈ 600) required sacrificial PAN mats to achieve film formation. Even with this aid, the resulting BPs exhibited larger aggregate domains, lower porosity, reduced flexibility, and decreased electrical conductivity and EMI shielding effectiveness compared to those of BPs made from shorter S-MWCNTs.

In contrast, S-MWCNTs (AR ≈ 158) enabled the formation of homogeneous, flexible, and highly conductive BPs even without the use of sacrificial supports. These BPs exhibited the highest surface area, superior intertube connectivity, enhanced AC electrical conductivity (>10^–1^ S/cm), and exceptional electromagnetic shielding (up to 36 dB in the X-band), despite their submillimeter thickness.

The use of sacrificial PAN mats, while necessary for certain nanotube geometries, was shown to introduce polymer residues that may hinder the electrical performance by disrupting the CNT network.

Overall, the findings indicate that shorter MWCNTs with moderate aspect ratios are more suitable for the production of structurally uniform and functionally superior buckypapers, particularly when aiming for enhanced electrical and EMI shielding performance. These results provide valuable guidance for the rational selection of CNT geometry and processing strategies for the development of next-generation buckypaper-based devices.
